# Antigen targeting to dendritic cells: Still a place in future immunotherapy?

**DOI:** 10.1002/eji.202149515

**Published:** 2022-06-02

**Authors:** Patrizia Stoitzner, Nikolaus Romani, Christoph Rademacher, Hans Christian Probst, Karsten Mahnke

**Affiliations:** ^1^ Department of Dermatology Venereology, and Allergology Medical University of Innsbruck Innsbruck Austria; ^2^ Department of Microbiology Immunology and Genetics University of Vienna Vienna Austria; ^3^ Institute of Immunology University Medical Center Mainz Mainz Germany; ^4^ Research Center for Immunotherapy (FZI) University Medical Center Mainz Mainz Germany; ^5^ Department of Pharmaceutical Sciences University of Vienna Vienna Austria; ^6^ Department of Dermatology University Hospital Heidelberg Heidelberg Germany

**Keywords:** Antigen targeting, C‐type lectins, Dendritic cells, Immunotherapy, Vaccination

## Abstract

The hallmark of DCs is their potent and outstanding capacity to activate naive resting T cells. As such, DCs are the sentinels of the immune system and instrumental for the induction of immune responses. This is one of the reasons, why DCs became the focus of immunotherapeutical strategies to fight infections, cancer, and autoimmunity. Besides the exploration of adoptive DC‐therapy for which DCs are generated from monocytes or purified in large numbers from the blood, alternative approaches were developed such as antigen targeting of DCs. The idea behind this strategy is that DCs resident in patients' lymphoid organs or peripheral tissues can be directly loaded with antigens in situ. The proof of principle came from mouse models; subsequent translational studies confirmed the potential of this therapy. The first clinical trials demonstrated feasibility and the induction of T‐cell immunity in patients. This review will cover: (i) the historical aspects of antigen targeting, (ii) briefly summarize the biology of DCs and the immunological functions upon which this concept rests, (iii) give an overview on attempts to target DC receptors with antibodies or (glycosylated) ligands, and finally, (iv) discuss the translation of antigen targeting into clinical therapy.

## Graphical abstract

Dendritic cell‐based immunotherapy is a promising vaccination strategy to treat infectious diseases, autoimmunity, and cancer. In this review, we present an overview on the current state of antigen targeting approaches via C‐type lectin receptors, with an outlook on future developments and potential for clinical translation.

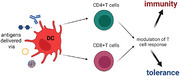

## Introduction

### Harnessing dendritic cells for immunotherapy

DCs were discovered by Ralph Steinman back in 1973 [1]. This, and the broad and sustained follow‐up research, including the introduction of DCs into immunotherapy of cancer, earned him the Nobel Prize in Physiology or Medicine 2011—“*for his discovery of the dendritic cell and its role in adaptive immunity*,” as the Nobel Committee worded it.

The outstanding capacity of DCs to elicit primary immune responses together with methods that allowed the relatively convenient generation of large numbers of DCs from monocytes of human blood [2, 3] soon led to their use in cancer immunotherapy [4]. Such clinical trials have been performed over the last two decades. These first‐generation DC vaccines were generated from patients' monocytes loaded with tumor antigens. When these monocyte‐derived DCs (moDCs) were reinfused into melanoma patients, activation and expansion of tumor‐specific T cells could be measured [5]. Many clinical trials followed over the years, testing different formulations of antigens (peptides, proteins, mRNA), different routes of administration (intradermal, subcutaneous, intravenous, intratumoral), and different ways to mature DCs (cytokine cocktails, TLR ligands) [6]. Nevertheless, only a small percentage of patients benefited substantially from adoptive DC‐therapy—in spite of mostly measurable immunological responses in vitro [7, 8]. Moreover, vaccine preparation at the scale needed for treatment is laborious. This is one of the reasons, why in the mid‐90s, Ralph Steinman's group came up with the idea that DCs resident in patients' lymphoid organs or peripheral tissues, such as skin, could be directly loaded (“targeted”) with antigens in situ [9].

In spite of the high potential of this elegant concept (that we will describe in more detail in this review), the thorough investigation of clinical applications was “overrun” by the success of checkpoint inhibitor therapies: *Science's* “breakthrough of the year” in 2013 [10]. DC‐based therapies appeared to have fallen into oblivion. This impression was not correct, though. Important studies and trials continued to be performed [11, 12, 13] and the urgent need for better immunization against cancer, that is, vaccination, is still present. Even the most recent evaluations of immune checkpoint therapies still document a not entirely satisfactory outcome due to primary or acquired resistance [14]. Foremost, still about half of the melanoma patients do not respond to immune checkpoints, emphasizing the need for improved immunization—thus, “antigen targeting” of DCs in situ being one of the most promising approaches to synergize with and complement immune checkpoint blockade. Aside from that, targeting antigens to DCs in situ may substantially improve vaccines.

### The history of antigen targeting to DCs—how did this idea develop?

The hallmark of the immunologic functions of DCs is their potent and unique capacity to activate naive resting T cells and, thus, initiate primary adaptive immune responses [15]. This view prevailed over a long time, beginning with Ralph Steinman's early and seminal studies that had established these defining features [16, 17]. Besides this mainstream theory, evidence has accumulated about a potential role of DCs in the generation or maintenance of peripheral tolerance [18, 19]. It soon became clear that the crucial checkpoint in the decision “immunity vs. tolerance” was the maturation of DCs. Mature DCs, equipped with costimulator molecules like CD80 or CD86 activate T cells and induce all their functions like cytokine production and cytotoxicity. Immature or semimature DCs, in contrast, lacking costimulator expression, can anergize T cells [20]. Over the past years, this almost black‐and‐white distinction has become more detailed and differentiated. Still, genome and transcriptome analyses do recognize pronounced and characteristic differences, with immunogenic DCs being regarded as having undergone “inflammatory maturation” [21] or “infection/inflammatory activation” [22] in contrast to tolerogenic DCs having been subject to “steady‐state maturation” [21] or “tolerogenic activation” [22]. Back at the end of the past millennium, however, it was difficult if not impossible to study the mechanisms of stably immature DCs experimentally. Why?

Virtually all methods to isolate DCs from tissues needed manipulations that would inevitably set off the maturation program of DCs. In skin, particularly in the epidermis, this was brought about by trypsinizing the tissue and subsequently mechanically “teasing” the cells apart to produce a single‐cell suspension. The epidermal Langerhans cells (LCs), literally “teased” in that way, would mature over some 2–3 days by default [17, 23], unless deprived of all survival cytokines in the case of highly purified populations [24, 25]. Similarly, the enzymatic treatment with collagenase and subsequent mechanical “grinding” of murine spleens or thymus, induced the maturation program in the low‐density cells so that after overnight culture, the resulting DCs were mature [26]. At that time, maturation was defined on the basis of morphology in phase contrast (cytoplasmic processes, “veils,” “hairy” appearance in the hemocytometer), phenotype (very high MHC‐class II expression, costimulator molecule expression), and function (very high stimulator function for allogeneic resting T cells in the mixed leukocyte reaction/MLR).

The investigation of the then novel group of C‐type lectin receptors (CLRs) [27] fed back on the above‐mentioned problem of studying immature, steady‐state DCs without kicking off the maturation or activation program. After having discovered and molecularly characterized the CLR DEC‐205 (CD205) in 1995 [28], it was the work of Mahnke et al. that hypothesized for the first time that “*DEC‐205 is used by DCs to improve antigen presentation*.” This was in the year 2000, and it was based upon the observation that an antigen was markedly better presented to antigen‐specific T cells when it was directed (“targeted”) to DEC‐205 on the surface of DCs, in side‐by‐side comparison with the mannose receptor (MR) [29]. It was not envisaged yet, to harness this for therapy or vaccination. This important study, however, introduced an elegant way to deliver an antigen in vivo to a defined DC subset (i.e., DEC‐205^+^ DCs) in a nondisruptive way that did not induce maturation.

This led to a first set of seminal papers in the years 2001 to 2005 that laid the foundation for the concept of “antigen targeting.” These studies, all done in mouse, revealed a robust and massive augmentation of T‐cell responses in the presence of DC‐activation stimuli [30, 31, 32] as well as of antibody production [33]. Targeting of antigen to various human DC surface receptors demonstrated that this concept has the potential to be translated into patients [34, 35, 36, 37, 38].

**Figure 1:**
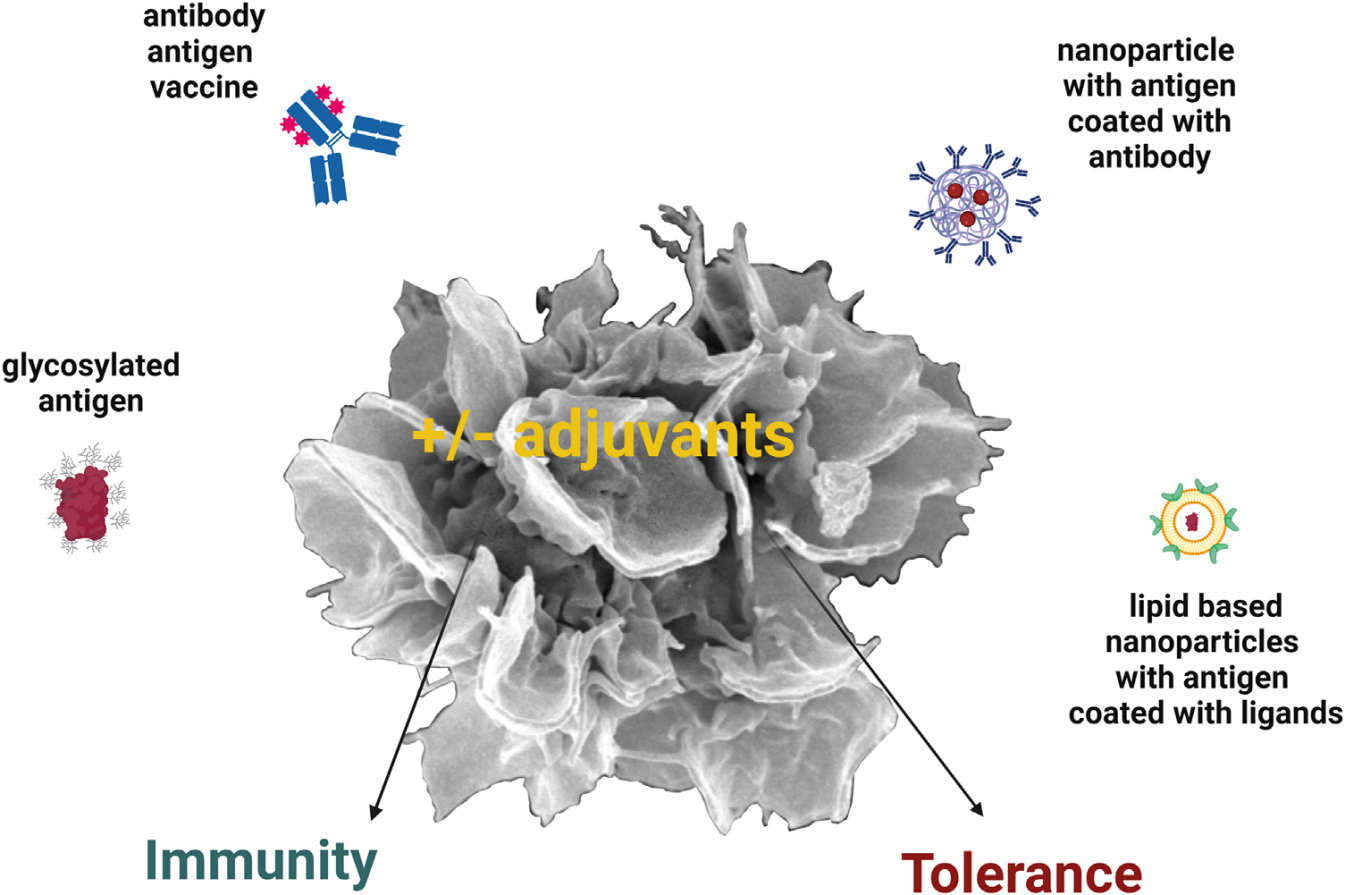
**DC‐based targeting approaches leading to immunity or tolerance**. Antigens can be delivered to DC in multiple ways as illustrated here. Antigens can be chemically or genetically conjugated to DC‐specific antibodies expressed on the surface of DC subsets, see Tables 1 and 2 for an overview on potential target receptors. Alternatively, glycosylated antigens can bind to and be taken up by C‐type lectin receptors which is a less specific targeting approach as these receptors can bind multiple sugar moieties. The development of nanocarriers, such as lipid‐based nanoparticles (LNPs), coated with either ligands or antibodies against DC‐specific receptors allows the encapsulation of antigens together with adjuvant for efficient delivery and routing inside the cells. The optimal formulation and delivery routes for nanovaccines need to be studied in the future to optimize DC‐based therapy to induce either tolerance (in the absence of adjuvants) or immunity (in the presence of adjuvants) for the treatment of autoimmunity or infectious diseases as well as cancer. Created with Biorender.com.

Still, the DC‐targeting strategies are in their infancy in humans, although, the first clinical trials targeting DEC‐205^+^ DCs have already been performed [39]. In this review, we will now highlight the most important developments of DC‐based targeting approaches (see Fig. 1 for a schematic overview on the diverse approaches).

## Why are DCs “perfect” targets for antigen loading in vivo?

Targeting of antigens to DCs via surface receptors for therapeutic use has by now been investigated for more than 20 years. The rationale behind this approach relies on the unique capacity of DCs to initiate adaptive immunity by priming of naïve T cells [15, 40]. Moreover, DCs can bridge the gap to the innate immune system by the secretion of IL‐12 and type I IFNs, as well as by mobilizing NK cells [41, 42].

Underlying the nonredundant role of DCs in T‐cell priming, is the ability of DCs to provide all three signals required for the priming and differentiation of T‐cell responses. These three signals are the presentation of antigenic peptides in complex with MHC class I and class II molecules (Signal 1), the surface expression of costimulatory molecules (Signal 2), and the secretion of cytokines required for the functional differentiation of T cells (Signal 3). DCs express high levels of MHC class I and MHC class II molecules, enabling them to prime both CD4^+^ and CD8^+^ T‐cell responses (Signal 1). Importantly, a subset of classical DCs termed conventional dendritic cells type 1 (cDC1) has the ability to feed antigens taken up through endocytic pathways into the MHC class‐I presentation pathway, and thus, prime CD8^+^ T cells against antigens expressed outside of the DCs itself, a process called cross‐priming [43]. Upon activation through signals, such as the recognition of pathogen‐derived molecules, inflammatory cytokines, or hallmarks of cell death, DCs upregulate the expression of CD80 and CD86 which interact with CD28 on naïve T cells, providing a costimulatory signal that is indispensable for T‐cell priming (Signal 2) [44]. Moreover, activated DCs provide additional signals via cytokines, such as IL‐12 and type 1 IFNs, or surface molecules, such as CD70 and OX40L to the primed T cells, which regulate their proliferation, differentiation, and survival (Signal 3) [45, 46]. Yet another unique DC feature, that renders them perfect targets for antigen loading *in vivo*—as opposed to macrophages, for example—is their capacity to migrate from the sites of antigen uptake (e.g., skin, muscle) to the sites where immune responses are started (T‐cell zones of lymph nodes). Thus, the migratory capacity of DCs is a prerequisite for their immunologic function, be it the induction of immunity or tolerance [47, 48].

With these features that define the unique potency of DCs for T‐cell priming emerging, ex vivo antigen loaded DCs have quickly been subjected to cellular therapy in various cancers, however, they did not turn out to be the “magic bullet”[49]. Despite showing comparable efficacy as CTLA‐4/ipilimumab checkpoint therapy in melanoma patients [8], efficacy needs to be further improved in the future [50]. One of the main setbacks of these novel therapies was that DCs from a patient's blood have to be prepared under GMP conditions, which makes the approach very expensive and time consuming. To avoid this personalized approach, in vivo loading with antigens by means of antibody targeting was envisioned as the next step [49, 51].

## General considerations for antibody‐mediated targeting of DCs

For antibody‐mediated targeting, beyond the challenges of choosing the right targets and antigens as well as developing methods for antibody:antigen coupling, the biological features of DCs in vivo have to be taken into account.

Early on, “DCs” was a general term for cells with dendritic shape and antigen presentation function but detailed analyses have revealed several lineages and different phenotypes (Table 1). Today, the most common scheme distinguishes cDC1, cDC2, plasmacytoid DCs (pDCs), moDCs, and LCs [48, 52]. Therefore, when choosing a surface molecule for targeting DCs, its expression pattern by the different subsets has to be taken into account, as it may influence the immunologic outcome. For example, targeting of molecules to CLEC9A, Langerin and DEC‐205 may lead to cross‐presentation by cDC1, whereas the DCIR‐1 surface molecule is rather specific for cDC2 targeting [53]. However, as DCs become more and more diverse, with different functionally and spatially distributed subsets residing in various tissues [52] there may be no “one fits all” DC‐targeting molecule after all.

**Table 1:** Overview on DC subsets and surface receptors for targeting approaches

	cDC1 (type I conventional DCs)	cDC2 (type II conventional DCs)	pDCs (plasmacytoid DCs)	MoDCs (monocyte‐derived DCs)	LCs (Langerhans cells)
Function	Cross‐presentation and priming of CD8^+^ T cells against extracellular antigens	Strong CD4^+^ T‐cell priming but also CD8^+^ T‐cell responses; most numerous subset in blood and tissues	Major producers of type I interferons upon virus infection	Develop from monocytes in vivo and in vitro during inflammation	antigen presentation to CD4^+^ and CD8^+^ T cells; role in tolerance induction
Surface molecules for targeting approaches	Clec9A/DNGR‐1 DEC‐205 XCR1	Clec10A/MGL Clec4a2/DCIR‐1 Clec7A/dectin‐1 DEC‐205 MR/CD206	Clec4C/BDCA‐2 DCIR‐1	DC‐SIGN/CD209 MR/CD206 DEC‐205 DCIR‐1	Langerin/CD207 DEC‐205

Besides the choice of antigen‐targeting receptors due to their expression patterns on DC subsets, the targeted receptors also need to mediate either active or passive endocytosis [27, 54]. In fact, the type of DC‐receptor targeted determines how antigen is taken up and intracellularly routed into endosomes and lysosomes [55]. Once internalized, the antigen must not be delivered to an exclusively degradative compartment (i.e. lysosomes), ideally the antigen attached to the targeting antibody gains access to MHC class II loading compartments for MHC class II presentation. In addition, DCs can deviate internalized antigens toward MHC class I presentation, a process called cross‐presentation [43]. For this, internalized antigens can be transported via the vacuolar pathway or via the endosome‐to‐cytosol pathway. In the vacuolar pathway, antigens are degraded in specialized endosomes containing the protease cathepsin S and MHC class I complexes. In these endosomes, MHC class I:peptide complexes are formed and delivered to the surface of the DCs. The endosome‐to‐cytosol pathway relies on the transport of antigens from endosomes into the cytoplasm, presumably by sec61/p97 protein complexes [56]. Once in the cytoplasm, the antigens are degraded by the proteasome. The resulting peptides gain access to the endoplasmatic reticulum (or go back to antigen‐loaded endosomes) via the TAP transporter and are finally loaded onto—and presented by—MHC class I molecules. A completely different pathway of MHC class I presentation is facilitated by cross‐dressing [57]. Here, complete MHC class I:peptide complexes are taken up by DCs and are integrated into their surface for stimulation of CD8^+^ T cells.

In murine lymphoid tissue, the model antigen ovalbumin (OVA) is mainly cross‐presented by the cDC1subset [58, 59] and in other organs, such as lung, intestine, and skin, the migratory XCR1^+^CD103^+^ DCs are stimulators of CD8^+^ T cells, making the cDC1 subset the major cross‐presenting cell type [60, 61]. However, other DC subpopulations have been shown to cross‐present as well [62, 63, 64]. Thus, cross presentation and “conventional MHC class II presentation” are not mutually exclusive processes. Many DC subsets are able to serve both antigen presentation pathways and the usage of different surface receptors may be critical for decision making. For example, antigen delivery to blood CD141^+^ cDC1 by targeting DEC‐205 might intracellularly route the antigen to early endosomes or lysosomes, whereas by targeting the CD40 molecule delivers cargo to early endosomes, favoring cross‐presentation [65].

Even more, the binding site of the targeting antibody is crucial. For instance, antibodies against the CLR DC‐specific ICAM‐3 grabbing nonintegrin (DC‐SIGN) can either detect the carbohydrate recognition domain (CRD) or neck region of the receptor. Although both antibodies bind to DC‐SIGN with the same efficiency, they differed in their way to mediate endocytosis and had different intracellular routing pathways, eventually leading to diverting T‐cell activation [66].

Although initially defined as stimulatory cells, DCs not just spur immunity, but they are also capable of inducing tolerance [67]. This feature is directly connected to their maturation or activation status, that is, immature non‐activated DCs can induce T‐cell anergy and regulatory T cells (Tregs). Therapeutic antibody targeting of DCs in situ has to consider that tissue‐residing DCs per se are not activated and, therefore, are rather tolerogenic [20]. Thus, when targeting antigens to DCs in vivo, one has to co‐administer activating stimuli, such as TLR ligands (i.e. poly I:C) and/or engage costimulatory molecules (i.e. CD40) for the induction of immunity [32]. In contrast, when antigen targeting aims at tolerance induction, the antibody:antigen conjugates must not disturb the “steady state” of tissue‐residing DCs to warrant tolerance induction [30, 68, 69]. This will be discussed in more detail in the context of DC‐targeting later in this review.

With these prerequisites in mind, some DC‐specific receptors have been identified and were further used for targeting approaches (for overview, see Table 2 and recent reviews [70, 71]).

**Table 2:** Overview of CLRs used for targeting of antigen to DCs

**Receptor**	**Expression pattern** **& possible ligands**	**Immunologic outcome after targeting**
DEC‐205/CD205 (29‐35, 37–39, 47, 65–78, 82, 84, 85, 90, 94, 95, 97, 115, 118, 134–136, 138–143)	Expression by cDC1, cDC2, LCs, pDCs, and sparsely by B cells in humans;Closely related to MR Ligands: Apoptotic/necrotic cells, binding via keratins, CpG‐oligonucleotides	‐ CD4^+^ and CD8^+^ T‐cell responses as well as humoral responses are induced but immunity requires concomitant activation of DCs by adjuvants ‐ Mediates effective cross‐presentation after coupling of tumor and pathogen‐derived antigens → immunity against tumors and infections ‐ Antigen presentation by steady‐state DCs in situ leads to tolerance induction by anergy and/or regulatory T cell activation → control of autoimmunity ‐ Single chain fragment variables are available for targeting of fusion proteins ‐ Clinical trials with DEC‐205‐antibody conjugated antigens were performed and demonstrated humoral and cellular immunity against HIV and melanoma
MR/CD206 (29, 38, 55, 56, 100, 101, 144–147)	Expression by macrophage populations, moDCs and blood CD1c^+^ DCs, but also expressed by non‐antigen presenting cells such as endothelial cells. Ligands: Generally glycoproteins belonging to mannosylated /*N*‐acetyl‐glucosamine–terminal and fucosylated neoglycoproteins.	‐ MR ligands were firstly used for targeting approaches ‐ Mainly used as target for antigen:ligand conjugates, data on antibody‐mediated targeting are sparse ‐ Preferentially guides antigens to the cross‐presentation pathways and induces CD8^+^ T‐cell activation → tumor immunity ‐ Ligand‐coated nanoparticles, such as liposomes and dendrimers loaded with specific cargo, have successfully been used to induce tumor‐specific T cells ‐ Clinical trials have been performed with oxidized mannan ligands and antibody‐conjugated tumor antigens → humoral and cellular responses were induced against adenocarcinoma and breast cancer
DC‐SIGN/CD209 (53, 58, 59, 124–126, 128, 148–154)	Expression by moDCs and dermal CD14^+^ cells Ligands: Recognizes Lewis‐type antigens and high mannose carbohydrates	‐ Ligand‐coated antigens (i.e. mannose or Lewis‐type glycans) and glycoliposomes decorated with Lewis‐type glycans target antigens via DC‐SIGN into human DCs ‐ antibody:antigen conjugates are taken up by human moDCs and generate T‐cell immunity ‐ DC‐SIGN targeting mediates CD4^+^ and CD8^+^ T‐cell responses ‐ human‐DC‐SIGN transgenic mice, humanized mouse models, and non‐human primates were used to prove translational potential for DC‐SIGN‐targeting as tumor therapy
Dectin‐1/CD369 (111, 112, 135, 155–160)	Expression by mouse cDC2s and human monocytes, macrophages and DCs Ligands: Primary pattern recognition receptor for glucans	‐ Modifications of antigens by natural Dectin‐1 ligands induce immune responses ‐ Dectin‐1 ligand has also been used as an adjuvant due to its ITAM engaging function → promising tool to enhance efficacy of other (targeted) therapies ‐ strong CD4^+^ T cell and B‐cell responses, stimulation of specific Th17 cells ‐ CD8^+^ T‐cell responses are weaker as compared to DEC‐205 targeting
Langerin/CD207 (58, 67–69, 72, 95, 113–115, 118–121, 161, 162)	Expression by mouse and human LCs, but also by mouse dermal cDC1 and splenic cDC1 (only in BALB/c mice) Ligands: mannose, N‐Acetylglucosamine (GlcNAc) and fucose	‐ Antibody‐mediated targeting of Langerin stimulated functional CD4^+^ and CD8^+^ T‐cell responses but also humoral responses ‐ Different T‐cell responses induced by dermal cDC1 and LCs in mice → LCs tolerogenic role, whereas dermal cDC1 primed T cells ‐ Skin vaccination studies prove potential to load LCs in situ ‐ ligands coating liposomes (i.e. Lewis Y or novel glycomimetic ligand) allow delivery of antigens to human LCs → resulting in cross‐presentation to CD8^+^ T cells
DCIR2/CD367 (36, 65, 73, 135, 163)	Expression by mouse cDC2 and human pDCs Ligands: binds to *N*‐glycan with bisecting GlcNAc and a GlcNAc‐terminated α1‐3 branch.	‐ Targeted antigens preferentially stimulate CD4^+^ T cells but also T cell‐dependent B‐cell responses, low activation of CD8^+^ T cells ‐ in humanized mice vaccination with DCIR‐specific antibodies stimulated T cells ‐ DCIR contains an ITIM motif and may have suppressive effects on antigen targeting, thus, maybe useful for tolerance induction
CLEC9a/DNGR1/CD370 (69, 76, 103, 105–110, 164–168)	Expression by mouse and human cDC1 Ligand: binds to actin on dying cells	‐ Targeting to CLEC9a by antibody:antigen conjugates induces strong antitumor immunity via the activation of CD8^+^ T cells, partially also CD4^+^ T‐cell responses ‐ Induction of strong humoral responses, which could be induced without adjuvant in mice and non‐human primates. However, it seems crucial to determine which antibody is used for targeting as not all induce a full‐blown immune response.

## Targeting surface receptors by glycosylation of antigens

Several targeting receptors belong to the family of CLRs and are selectively expressed by subtypes of DCs. This family of receptors binds complex oligosaccharides on cell surfaces and glycoproteins in blood and other biological fluids. They contain independently folding, modular CRD that bind sugars by ligation to Ca^2+^. In particular, in the immune system, many receptors (e.g. E‐, P‐, l‐selectin, asialoglycoprotein receptor, Fcε receptor CD23) belong to the CLR family and fullfill functions in cell adhesion, glycoprotein turnover, or pathogen recognition [72]. Therefore, CLRs make up a natural target for in‐vivo loading of DCs [27]. To render antigen targeting specific for these types of receptors, initial approaches took artificially glycosylated antigens, whereby defined sugar moieties were expected to warrant specific binding [73].

For the MR, whose ligands are readily defined (i.e. mannose, fucose), mannosylation of antigens leads to a 10,000‐fold higher presentation by MHC class II molecules as compared to non‐mannosylated [74]. The cross‐presentation of glycosylated antigen was even more enhanced after conjugation to oxidized mannan, as compared to antigens being conjugated to reduced mannan [75]. Still, specific targeting of the MR^+^ DC subsets by glycosylated ligands remains questionable. Though all CLRs indeed express different CRD domains, they may still have overlapping specificities for glycosylated ligands. Therefore, mannosylated antigens may also bind to other CLRs on DCs or other myeloid cells.

“Sugar coatings” using the difucosylated oligosaccharide  Lewis (Le)Y have also been studied for antigen targeting approaches to the CLRs DC‐SIGN and Langerin, which are expressed by defined human and murine DC subsets [27]. Conjugating LeY to peptides or antigen‐loaded liposomes led to uptake and antigen presentation by human LCs and dermal DC‐SIGN^+^ CD14^+^ myeloid cells to various degrees. LeY‐modification of liposomes induced binding to LCs, but the presentation of antigens to CD8^+^ T cells was not enhanced. In contrast, after being taken up by DC‐SIGN^+^ CD14^+^ myeloid cells, cross‐presentation and activation of CD8^+^ T cells followed. On the other hand, when peptide antigens were modified by LeY‐efficient cross‐presentation was only achieved by LCs, whereas SIGN^+^ CD14^+^ myeloid cells failed to do so [76]. In line with this, the group of van Kooyk demonstrated that LeX‐ and LeY‐modified antigens showed differential binding, antigen‐presentation and T‐cell stimulation via multiple receptors, such as DC‐SIGN and macrophage galactose‐type lectin receptor 1 [77, 78].

From these studies, it can be concluded that glycosylation of antigens to target CLRs is an interesting alternative to antibody‐mediated DC‐targeting, and thus, should be considered for future vaccination strategies [79]. However, as differential uptake by CLRs on DCs and macrophages influences the nature and strength of the immune responses, one needs to carefully evaluate how glycosylated antigens are internalized and processed by DCs and other myeloid cells.

## Targeting DCs with antibodies to prototypic surface receptors

Among the first promising surface molecules for DC‐targeting approaches, DEC‐205 and the MR were identified [29]. These receptors have been used as prototypic targeting receptors for understanding the basic principles of DC‐targeting on immunity and tolerance and are discussed in more detail. Beyond DEC‐205 and MR, other receptors are also investigated as discussed later in this review and are summarized in Table 2.

To begin with, MR and DEC‐205 belong to the CLR family and are naturally involved in antigen uptake of ligands derived from bacteria, yeast, and viruses, as well as certain endogenous glycoproteins [27]. In vitro cultures of DC revealed that both receptors take up antibody:antigen conjugates with high efficiency and direct the antigens through the endosomal processing machinery for presentation through MHC class II and I (cross‐) presentation pathways [29]. Both targets can enhance antigen presentation 100‐ to 1000‐fold in vitro as compared to unconjugated antigens [31].

Although MR and DEC‐205 are 95% homologs by amino acid (AA) sequence, placing both of them into the type I group of CLRs, some fundamental differences exist. The DEC‐205 receptor contains ten CRD domains, extending the number of possible ligands even more, whereas the MR harbors only eight. Even more importantly, the intracellular domain of DEC‐205 is highly unique among all CLRs as it contains a defined AA sequence with four acidic amino acids, enabling the receptor en route targeting to late endosomal MHC class II^+^ compartments and recycling back to the surface. These features prevent endocytosed antigens from deviating to proteolytic pathways, and the repetitive recycling of the receptor from surface to MHC–II loading compartment (MIICs) and back allows import of huge amounts of antigens [29]. As compared to the MR, which is expressed by some DC‐subtypes, macrophages and also liver cells, DEC‐205 expression is largely restricted to DCs; nevertheless, some expression by B cells has been detected, too [80]. Although the natural ligands are ill defined, binding of apoptotic and necrotic cells for uptake and cross‐presentation of debris‐associated antigens to T cells has been demonstrated [81].

As DEC‐205 is the most used and best‐studied CLR for DC‐based targeting therapy, we will discuss this strategy in more detail below.

### Targeting surface receptors of DCs to induce immunity—The DEC‐205 example

Generally speaking, the main intrinsic function of DCs is to interact with T cells for the induction of tolerance or immunity. In contrast, MR^+^ macrophages can mostly be found in situations that require their scavenging function (atherosclerotic plaques, bacterial infections) or induction of a less fulminant immune reaction [82]. Therefore, antigens taken up by DEC‐205 in DCs are less likely to be fully degraded and more likely to be presented as peptides to T cells as compared to MR targeted antigens, making DEC‐205 an ideal candidate for antibody targeting [29]. As the ligand(s) for DEC‐205 still remain elusive, targeting experiments were done with antigens fused to anti‐DEC‐205 antibodies.

Various antigens were coupled to DEC‐205 antibodies, and the efficacy of these novel vaccines was studied in murine and human DCs. Multiple studies demonstrated that antigen is efficiently incorporated into various murine and human DC subsets via DEC‐205 and subsequently boosted T‐cell responses in vitro and in vivo [31, 34, 35, 37, 38, 53, 83‐86]. Using DEC‐205 as target, antigens induced CD8^+^ and CD4^+^ T‐cell and humoral immune responses with greater efficacy, when compared to nontargeted antigens [32, 84].

The potential of DEC‐205‐targeted approaches for immunotherapy was revealed by the evaluation of DC‐based vaccines in murine disease models. Specific delivery of melanoma antigens via the DEC‐205 receptor led to prevention or reduction of tumor growth [32, 87‐90]. In infection models, DEC‐205‐mediated delivery of pathogen‐derived antigens could prevent the development of pneumonic plague [91], induced HIV‐specific immunity [53, 92], or protected against influenza infection [93]. In addition to complete antibodies, even single‐chain variable fragments fused to different antigens or fusion with encoding DNA vaccines have been tested successfully [37, 94, 95]. This strategy was used in a mouse melanoma model, where the melanoma‐associated antigen gp100 delivered via DEC‐205 to DCs was superior in suppressing tumor growth than peptide vaccination [94].

The addition of adjuvants is required to induce immunity to cancer or pathogens, and ligands for pathogen‐recognizing receptors, such as TLRs, RIG‐I like receptors, and NOD‐like receptors, are used to give DCs a danger signal [96]. The most commonly used adjuvants are TLR‐ligands, such as poly I:C (TLR3/MDA5/RIG‐I), CpG (TLR9), or imiquimod (TLR7), often combined with an agonistic antibody against the costimulatory molecule CD40. Indeed, DEC‐205^+^ DCs respond exceptionally well to poly I:C stimulation leading to IFN‐production and subsequent DC activation with enhanced antigen presentation [97]. Targeting approaches with DEC‐205 antibodies could be clearly improved by the addition of TLR ligands [32, 87, 89, 98]. For example, only the combination led to tumor regression in respective models [32, 87‐90]. Moreover, novel adjuvants, such as the STING activator c‐di‐CMP, have been tested in a DEC‐205 targeted vaccination approach with superior results over traditionally utilized poly I:C and CpG [99]. An alternative approach followed by Seders’ group is conjugating antigens to TLR7/8 agonist to generate conjugate vaccines. The OVA‐protein delivered that way elicited potent CD4^+^ and CD8^+^ T‐cells responses of the Th1‐type [100].

And finally, site‐directed antibody engineering even allows to couple both, the antigen and the adjuvant (CpG or poly dA:dT) to DEC‐205 antibodies, leading to improvement of T‐cell responses, when compared to sequential injection of antibody:antigen conjugates and adjuvants [101, 102]. These triple conjugates of antibody:antigen:adjuvant may avoid side‐effects caused by bystander cell activation, as the adjuvant is locally bound by the antigen‐loaded DCs [103]. An alternative approach is encapsulating intracellular TLR ligands in nanoparticles coated with antibodies targeting DC receptors [104].

Therefore, in clinical settings, not just the antibody or the coupling of an antigen to the antibody matters for successful vaccination, but also the adjuvant of choice is crucial for the resulting immune response.

### Targeting surface receptors of DCs to induce tolerance—The DEC‐205 example

In contrast, using targeting of DEC‐205 without concomitant application of adjuvants leads to tolerance induction, as shown with the pioneering work by Ralph Steinman's lab [30, 31, 68]. Induction of peripheral CD4^+^ and CD8^+^ T‐cell tolerance as result of antigen presentation by steady‐state DCs was confirmed in mouse models transgenically expressing model antigens in DCs [105, 106]. While CD8^+^ T‐cell tolerance induction employs antigen‐specific repressive mechanisms, such as anergy or deletion of specific T cells [31, 105], tolerance induction upon presentation of MHC class II restricted model antigens was found to be at least partially dependent on the induction of Tregs [68, 106, 107]. However, using mice that allow depletion of FoxP3^+^ cells proved that Tregs are required to maintain the tolerogenic function of steady‐state DCs [108]. Indeed, mouse models lacking antigen presentation on MHC class II revealed that cognate interactions between Tregs and DCs are required to maintain peripheral CD8^+^ T‐cell tolerance and prevent spontaneous autoimmunity [109, 110].

The applicability of antigen targeting to steady‐state DCs as a means to prevent or ameliorate autoimmunity was subsequently demonstrated in preclinical animal models of autoimmune disease. Delivery of a beta‐cell autoantigen to DCs via DEC‐205 targeting resulted in the deletion of autoreactive CD8^+^ T cells in NOD mice, a mouse model of type I diabetes [111]. Targeting of MHC class‐II restricted epitopes from myelin oligodendrocyte gp [106, 112] or proteolipid protein [113] to DCs protected the mice against experimental autoimmune encephalomyelitis (EAE) elicited by injection of the respective autoantigen in adjuvant. Both mechanisms of tolerance, such as deletion of autoreactive T‐cell clones [113] and the induction of FoxP3^+^ Tregs [106, 112, 113], were shown to be involved in the protective effect of antigen targeting to DCs. This has further been confirmed for models of inflammatory bowel disease and arthritis [114].

In aggregate, the detailed analysis of DEC‐205 targeting in cellular and murine models has initiated several clinical and preclinical trials for vaccination with antibody:antigen conjugates, as discussed further below.

## Targeting alternative receptors

Mainly CLRs targeted by antibodies or ligands have been used so far for DC‐based immunotherapy. Despite DEC‐205, many other CLRs have been tested for their potential to boost T‐cell responses to targeted antigen as nicely summarized in Lehmann et al. [70] and Baldin et al. [71]. Several studies also compared different DC‐surface receptors with DEC‐205 in regards to induction of cellular immune responses. A summary can be found in Kastenmüller et al. [50]. We would like to briefly mention the most commonly used alternative surface receptors besides DEC‐205 for DC‐targeting approaches.

As mentioned earlier, the MR was one of the first potential targets identified to deliver antigen to DCs [29]. Most studies were performed with glycosylated antigens [74, 75], however, also antibody‐targeting approaches against MR were tested [115]. Tumor antigens were guided into the cross‐presentation pathway to induce CD8^+^ T‐cell activation [116, 117], but also led to CD4^+^ T‐cell responses [38]. In regards to clinical translation, this antibody against MR was conjugated to the human tumor antigen chorionic gonadotropin beta‐chain and induced consistent humoral and T‐cell responses when coadministered with TLR agonists in patients with advanced epithelial malignancies [118].

Due to their excellent cross‐priming capacity, targeting specifically the cDC1 subset was envisioned as an excellent strategy for cancer therapy. In this regard, an interesting surface receptor expressed only on cDC1 is Clec9A/DNGR‐1 [119]. Targeting of Clec9A induces priming of CD4^+^ and CD8^+^ T cells but also humoral responses [120]. A special feature of the Clec9A‐targeting approach is the induction of a potent B‐cell response ileading to strong antibody production even occurring in the absence of adjuvants [121, 122]. This could be successfully translated into the human situation, as CD141^+^ cDC1 can be targeted via Clec9A to boost antigen‐specific T‐cell responses [123, 124]. This strategy also looks promising for cancer immunotherapy supported by experiments using cancer antigens conjugated to Clec9A antibody [119, 125, 126].

Other targeting strategies have employed antibodies against dectin‐1 and Langerin, which have both been tested for their potential as skin vaccination approaches. Dectin‐1 is expressed on the dermal cDC2 subset and antigen delivered via this receptor leads to T‐ and B‐cell responses in mouse models [127]. This finding could be confirmed with human moDCs and dermal cDC1 that induced CD8^+^ T‐cell responses against melanoma and flu peptides [128]. Langerin functions as a pattern recognition receptor (PRR) on murine and human LCs, however, can also be expressed by murine cDC1 [129]. In mouse models, antigens targeted to Langerin are efficiently presented to CD4^+^ and CD8^+^ T cells by lymph node and spleen cDC1 [130] and induced anti‐HIV immunity [53] as well as strong cytotoxic CD8^+^ T‐cell responses in vivo [89]. However, Langerin targeting can also lead to tolerance induction by Tregs [89, 112]. Moreover, LCs can drive activation of follicular Th and promote humoral responses after Langerin‐targeting [131]. In experiments with human skin explant cultures, the potential of targeting LC with Langerin antibodies was confirmed [132], however, data on subsequent human T‐cell stimulation are still missing [133, 134]. First attempts to study T‐cell responses induced by human skin DCs targeted via Langerin, that is, LCs, yielded equivocal data that call for more research into this (technically and logistically difficult to work with) topic [135]. A recent development of a highly specific glycomimetic ligand for Langerin represents an interesting alternative to antibody targeting for the development of novel LC‐based vaccines [136, 137, 138].

Targeting of DCIR2 which is a more ubiquitously expressed CLR on multiple DC subtypes, caused mainly induction of CD4^+^ T‐cell activation and strong antibody responses [83, 90]. This CLR also mediated cross‐priming of CD8^+^ T cells in human DCs [36, 139].

As already mentioned earlier, DC‐SIGN has been employed in many human studies as targeting receptor, however, its expression is limited to moDCs and dermal CD14^+^ cells (formerly regarded as dermal CD14^+^ DCs) that reflect a macrophage‐like phenotype [140]. Antibody and glycan‐based targeting strategies proved the potential of DC‐SIGN to mediate CD8^+^ T‐cell responses [141, 142, 143]. There are murine homologs but their expression differs strongly from human DC‐SIGN [144], still, DC‐SIGN targeting in mice leads to strong T‐ and B‐cell responses [145].

Surface receptors not belonging to the CLR‐family have been studied for DC‐based immunotherapy. For example, XCR1 is a chemokine receptor expressed exclusively on cDC1 and when targeted by fusion proteins consisting of the chemokine XCL1 and antigens caused potent antitumor responses [146, 147, 148] as well as T‐ and B‐cell responses against influenza [93, 149]. The high expression of Fc receptors on DCs was employed by Diana Dudziaks’ group to deliver OVA protein to DCs leading to superior T‐cell responses when FcγRIV was used [150].

In summary, a multitude of different receptors, even on the same DC subtype, makes it a difficult choice which surface molecule should be targeted especially as they can influence the subsequent immune response. This implies and requires the careful choice of receptor that will determine the desired T‐cell response against infection and cancer.

## Translation into the clinics

The first clinical trials testing DC‐based immunotherapy were performed over two decades ago [4]. These first‐generation DC vaccines were generated from patients' monocytes and loaded with tumor antigens. When these moDCs were reinfused into melanoma patients, activation of tumor‐specific T‐cell responses could be measured [5]. Nevertheless, only a small percentage of patients benefit from DC‐therapy and vaccine preparation is laborious [7, 8].

As outlined above, the proof of the DC‐targeting principle came from mouse models and revealed that antibody‐mediated delivery of antigen to DEC‐205 on DCs leads to endocytosis of antigen. Subsequently, antigenic peptides were loaded on MHC‐class I and II molecules and T‐cell responses were efficiently boosted (we refer to excellent reviews [7, 50, 70, 71]). As said, most studies were performed in mice, and the first translational studies were done by using humanized mice. With these models, it could be confirmed that: (i) DEC‐205 induced Epstein Barr virus‐specific T‐cell responses in vivo [35, 151], (ii) Clec9A targeted antigens to CD141^+^ DCs [124], and (iii) DC‐SIGN induced T‐cell responses protective against *Listeria* infection [143]. A recent study compared several of these receptors, namely DEC‐205, DCIR, Dectin‐1, and CD40, to deliver influenza antigens to humanized mice, and indeed all of them were able to induce human CD8^+^ T cells in vivo. This study nicely points out that humanized mice are a suitable tool to investigate and further develop DC‐based targeting strategies before testing them in the clinics [152]. In support of this, in vitro studies with human DCs highlighted the translational potential of this DC‐based vaccination approach [34‐38, 141, 142].

Very few clinical studies have addressed the potential of DC‐based targeting approaches in the treatment of patients. One of the first steps to bring this strategy into the clinic was a randomized dose escalation study with healthy human volunteers receiving DEC‐205 conjugated to an HIV‐peptide subcutaneously together with the adjuvant poly‐ICLC. So far, it was reported that HIV‐specific antibodies were detected in the volunteers [153]. At the same time, another trial tested a vaccine composed of human chorionic gonadotropin beta‐chain conjugated to an anti‐MR antibody (CDX‐1307^TM^, Celldex Therapeutics) given either locally (intradermally) or systemically (intravenously) in patients with advanced epithelial malignancies. When used with the adjuvants poly‐ICLC and TLR7/8 agonist resiquimod consistent humoral and T‐cell responses were measured [118]. Subsequent trials of targeting the cancer germ line or testis antigen NY‐ESO‐1 coupled to an anti‐DEC‐205 antibody (CDX‐1401^TM^, Celldex Therapeutics), induced some humoral and cellular immunity in patients with solid tumors with no signs of toxicity; clinical response was not assessed [38]. This vaccine is currently being investigated in different combinations in clinical trials, for example, with immune checkpoint blockade antibodies. Recently, Bhardwaj et al. published results from a clinical study that revealed that by adding Flt3L to the anti‐DEC‐205‐NY‐ESO‐1,vaccine monocyte and DC numbers could be boosted, which correlated with increased humoral and T‐cell responses [154].

All these clinical data point at the promising potential of DC‐targeting strategies to boost T‐cell responses against pathogens and tumors, however, clinical data on patient outcomes are still scarce.

## Targeting mRNA vaccines to DCs

Antigen targeting to DCs may also be of high relevance with respect to the recent advances in mRNA technologies. First and foremost, the success of SARS‐Cov‐2 mRNA vaccines is based on delivering mRNA to DCs after intramuscular administration of nucleic acid encapsulated by lipid‐based nanoparticles (LNPs). This lipid shell promotes mRNA delivery to DCs and results in antigen secretion, processing and presentation, eventually eliciting a protective immune response [155]. Besides this considerable interest in prophylactic vaccination, DCs have also been targeted with mRNA for efficient cancer‐antigen presentation either ex vivo, followed by readministration of moDCs or LCs [156, 157, 158] or in patients using LNP‐formulations [159]. Moreover, delivering noninflammatory mRNA vaccines to lymphoid tissue‐resident CD11c^+^ APCs in a tolerogenic state in an EAE model has shown great promise for the treatment of autoimmune diseases [158].

However, when administering such LNP‐based vaccines via the preferred route, that is skin and muscle, some of the mRNA vaccine may also end up in sites irrelevant for immunization, such as ovaries and testes, as shown for the current SARS‐CoV‐2 mRNA [160]. Consequently, targeted delivery to DCs for reducing off‐target cells, would allow dose sparing and higher versatility of the mRNA technology. The uptake of LNPs is mediated by Apolipoprotein E, which then allows for the recognition of the low‐density lipoprotein receptor, expressed by the targeted cells. Likely, many DC subsets benefit from the high expression of this receptor [161]. The underlying mechanisms of LNP‐mediated uptake, specifically into DCs compared to other cells at the injection site, remain understudied and the DC‐specific features, such as endosomal routing, membrane composition, acidification, and escape of the mRNA to the cytosol, are not well understood, in particular, with respect to DC subset specificities and effectiveness.

Utilizing targeting moieties, such as antibodies conjugated to LNPs [162] or carbohydrates ligands [163], increases specificity and may even alter endocytic routing and escape mechanisms. In both cases, specific receptors expressed on DCs are necessary. Alternative approaches addressing this necessity, still promising highly effective DC delivery are based on altered lipid composition of the LNPs [164, 165]. In this respect, the skin might gain more attraction in the future as an alternative administration route for mRNA‐based vaccination, since long‐lasting mRNA expression in the skin has been previously shown compared to other routes [166], leading to prolonged, systemic antigen exposure [167]. Taken together, it can be expected that targeted delivery of the lipid particle‐enwrapped mRNA may improve immunization efficiency, reviving the question on which is the most effective DC subset in regards to induction of T cell responses. This focus may be brought about by “dressing” lipid particles with ligands for CLRs expressed on DCs [136] or with antibodies specifically binding to DCs.

## Conclusion and future perspectives

DC‐targeted therapies are a realistic hope for future therapies of cancer, infectious diseases, and autoimmunity [50] most likely in synergy with other immunotherapies. In the case of cancer, synergies can be expected when combining DC‐targeting with immune checkpoint blockade. Such a “two‐pronged” approach would both harness preexisting (antigenically undefined) tumor immunity in patients by releasing and amplifying it by means of checkpoint blockade, and in addition, generate de novo immune responses against cancer by means of targeting DCs with defined tumor antigen (“cancer vaccine”). DC‐targeting may be especially beneficial when patients mount insufficient immune responses against their tumors, as often reflected in tumors with no or few infiltrating immune effector cells [14]. Given the unprecedented rapid progress in biomedical technology, it is well conceivable that, at some point in the future, DCs may be targeted with defined (neo)antigens specific to and optimal for each patient [168]. While these hopes can ultimately be fulfilled, several topics still need to be thoroughly investigated to improve vaccine delivery (intradermal, subcutaneous, intraveneous), vaccine formulation (antibody‐antigen fusion proteins, CLR‐ligands conjugated to antigens, neoglycosylated antigens, antibody or ligand coated nanovaccines encapsulating antigen and adjuvants), and adjuvant type (poly I:C, STING agonists, etc.) to optimally activate DCs. And last but not least, there is still the question whether targeting one DC‐subset is more powerful than targeting a receptor expressed on multiple DC‐subtypes.

In closing, we would like to emphasize that the large body of evidence from mouse models, from human in vitro studies, humanized mice, and from the few first clinical attempts makes it worthwhile and ultimately rewarding to further investigate this topic with energy and enthusiasm.

## Conflict of interest

The authors declare no commercial or financial conflict of interest.

**Abbreviations**
cDC: conventional dendritic cells
CLR: C‐type lectin receptors
CRD: carbohydrate recognition
DC‐SIGN: dendritic cell‐specific ICAM‐3 grabbing non‐integrin
LC: Langerhans cells
Lewis: Le
LNPs: lipid‐based nanoparticles
moDCs: monocyte‐derived dendritic cells
MR: mannose receptor
OVA: ovalbumin
pDCs: plasmacytoid DCs
